# A Suspended Six-Port Transformer-Based Power Divider for 2.4 GHz Applications

**DOI:** 10.3390/mi8040118

**Published:** 2017-04-08

**Authors:** Wen-Hui Huang, I-Yu Huang, Wun-Hong Syu, Chia-Lung Sung, Chia-Hsu Hsieh

**Affiliations:** Department of Electrical Engineering, National Sun Yat-sen University, 70 Lienhai Rd., Kaohsiung 80424, Taiwan; d993010005@student.nsysu.edu.tw (W.-H.H.); iyuhuang@mail.nsysu.edu.tw (I-Y.H.); wei199008@gmail.com (W.-H.S.); dragon801022@gmail.com (C.-L.S.)

**Keywords:** transformer-based, power divider, six-port, suspending structure, wireless applications

## Abstract

This paper presents a transformer-based power divider with six-port suspending structure for 2.4 GHz wireless applications. The proposed power divider, which is featured with chip size (2.9 mm × 2.8 mm × 21 μm), was constructed by an 8-μm-thick Cu bottom electrode, a 5 μm-height supporting copper post, and an 8-μm-thick suspended spiral copper conducting layer with a 13 μm air gap. The main structure included two transformers and six input/output matching capacitors for simultaneously achieving two single-to-differential paths so that the chip size of the complex multiple-ports transceiver could be reduced. According to the results, the proposed divider has characteristics of the radio frequency (RF), and its input return losses are around −10 dB, output return losses are beneath −10 dB, and minimum amplitude imbalance is below 1.5 dB and less than 1° phase imbalance at 2.4 GHz operating frequency.

## 1. Introduction

**Figure 1:**
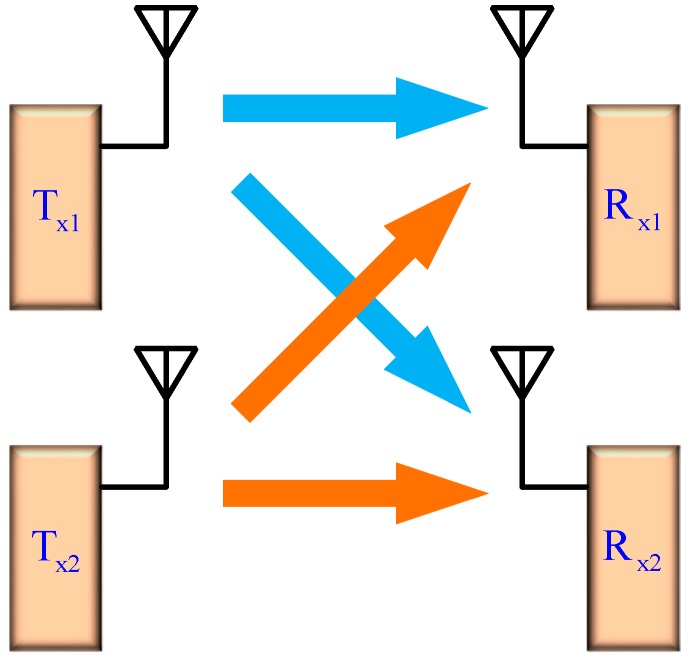
Diagram for the 2 × 2 multi-input multi-output (MIMO) transceiver.

Increased demands for high transmission speed for wireless communication system have led to the popularity of transceivers with in/quadrature (I/Q) architecture [1,2]. However, to achieve higher data rate, new designs of transceivers are necessary for applications such as industrial, scientific and medical (ISM) radio band (emerging low-power appliances), long term evolution (LTE) mobile networks, multimedia (video streaming, e-commerce), wireless internet access (WLAN, WiFi, WiMax) and wireless body area sensor networks (WBANs) with multi-input multi-output (MIMO) topologies [3]. Figure 1 depicts the diagram for the 2 × 2 MIMO transceiver [4], which comprises two receivers (Rx) and two transmitters (Tx), and both Rx and Tx path have the double conversion architecture [5]. The duplicate functional components are required, that is, passive items with compact size are important after the MIMO architectures are adopted in the transceiver.

Power splitter is an essential and critical component widely used in RF wireless systems [6,7,8,9,10,11,12] to provide balanced outputs from an unbalanced input for antenna array [6], or to reduce the reflected wave as an impendence matching network in low-noise amplifiers (LNAs) [7,8,9]. Power dividers also have been applied to mitigating image rejection [10] and improving voltage conversion [11] in mixers, and are often used in voltage-controlled oscillators [12] to enhance phase noise characteristic.

The reported power dividers [13,14,15,16,17,18,19,20,21,22,23] can be classified into active and passive types. Active power dividers usually use two common-drain FETs [13] or Darlington cell [14] topologies to achieve single-to-differential outputs by adopting advanced processes. Despite the advantage of compact size, active dividers are unsuitable for portable systems due to extra power dissipation. Passive dividers, on the other hand, are mostly realized in monolithic microwave integrated circuit (MMIC) technology [15,16,17,18] and are seldom performed in MEMS process [19] to split unequal power signal into differential. However, it is still a challenge to implement a power divider with miniaturized size by cause of the limitation of wavelength. Some techniques were proposed to reduce the size of power dividers, such as capacitive loading technique [20] and lumped components based on Method of Least Squares (MLS) technique [21], but they do not have multiple ports to simultaneously realize two differential outputs. So, six-port splitters were proposed using a Wilkson power divider combined with three quadrature hybrid couplers (or three branch-line couplers) [22], and a rat-race coupler incorporated with three branch-line couplers [23]. Although the mentioned splitters are suitable for I/Q transceivers, they are still too large to be integrated with RFIC chips.

**Figure 2:**
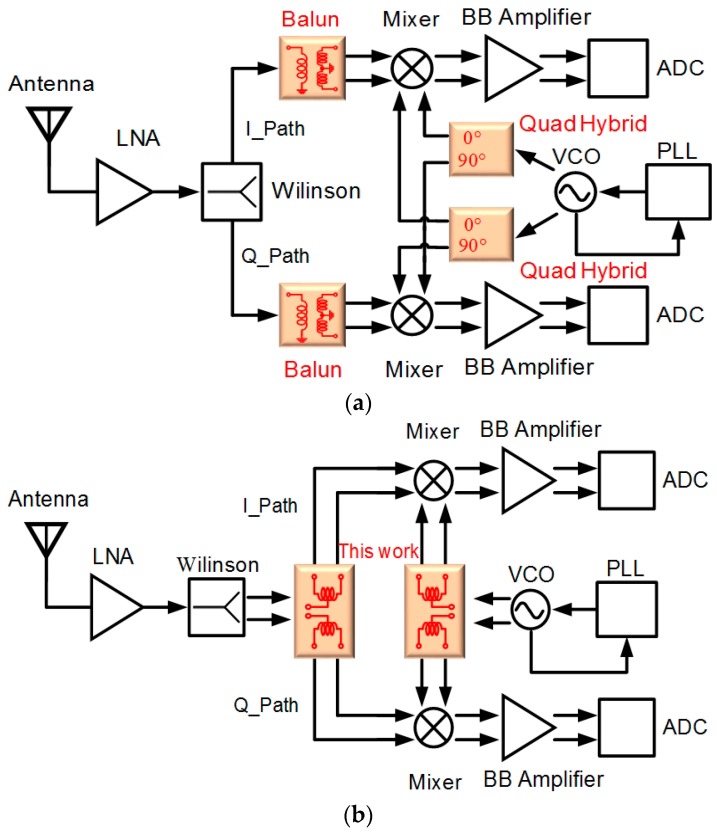
The receiver front-end architecture with: (**a**) conventional passive device (**b**) six-port transformer-based power divider.

In the receiver front-end architecture shown in Figure 2a, mixer is an indispensable component with differential configuration since it has better port-to-port isolation and noise immunity [24], and the passive blocks, such as single to differential baluns and 90° phase shifters, are required to realize the differential signal. However, passive blocks always dominate the entire receiver chip size, let alone the MIMO transceiver system. Figure 2b presents the implemented suspended six-port transformer-based power divider adopted in the receiver front-end, which provides two differential outputs for simultaneously reducing the passive blocks and the complicated multiple Rx or Tx MIMO chip dimension.

The implemented suspended six-port transformer-based power divider is featured with compact size and good phase imbalance for multiple components on silicon substrate utilizing surface micromachining technology. In the main structure, multilayer transformer configurations were used to shorten the dimension of the power divider, and the MIM capacitors located at input/output ports were used to improve the impedance match. The proposed power divider is suitable for multiple Rx or Tx MIMO applications. In the following sections, we will show the structure of the proposed six-port transformer-based power divider, the manufacture of fabrication processes, the measured results of divider, and its comparison with simulated performances.

## 2. The Structure of the Six-Port Power Divider

**Figure 3:**
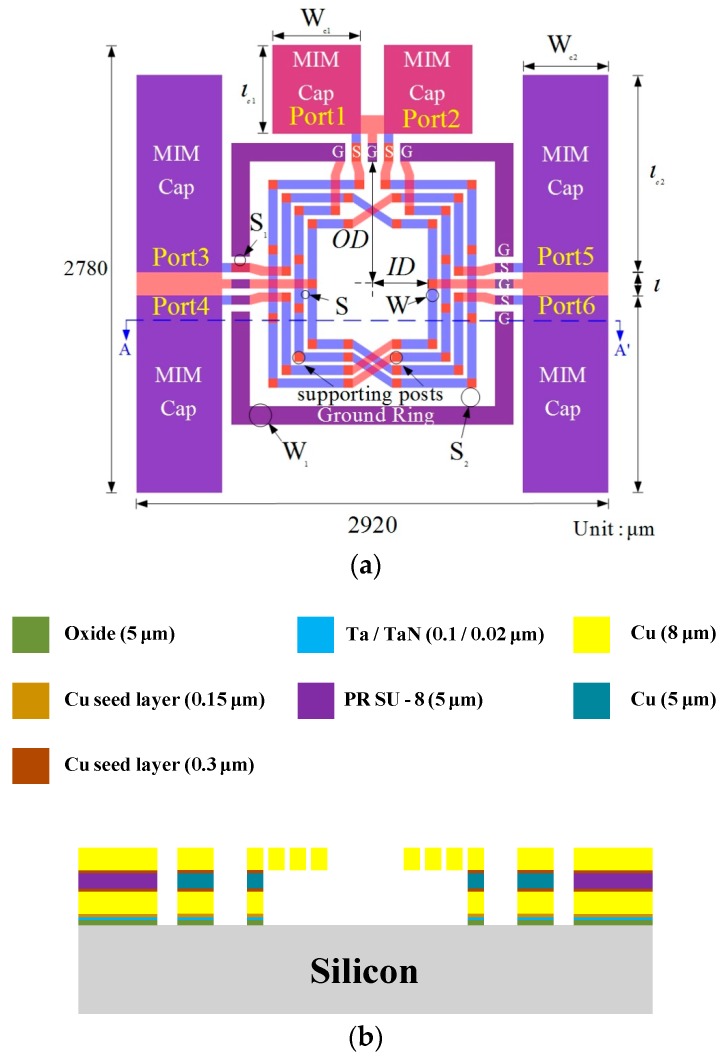
(**a**) Top and (**b**) cross-section views of the six-port transformer-based power divider.

The six-port transformer-based power divider consisted of two inner symmetric transformers, ground ring and six exterior metal-insulator-metal (MIM) capacitors (C), and the top and cross-section views of the divider is shown in Figure 3. Power incident at input port (port 1) was coupled to the output ports (port 3 and port 4), and the phases at port 3 and port 4 were 90° and −90°, respectively. Similarly, the power incident in port 2 was coupled to ports 5 and 6, and there were two 180° phase differences between each output ports (port 3 and 4, port 5 and 6). The layer thicknesses of power divider were calculated according to the skin effect equation [25], and the skin depth (δ) was defined as

(1) $$\delta =\sqrt{\frac{2}{\omega \cdot \mu \cdot \sigma }}$$

where ω is the angular frequency, µ is the permeability and б is conductivity, and the computed result is around 1.5 µm. Since thickness is usually five times higher than the skin depth for reducing conductor loss, the thickness of inner symmetric transformers has to be higher than 7.5 µm, and we set the thickness as 8 µm to avoid structure collapse while the devices were released. Free-standing symmetric transformers was also used to reduce the substrate loss with 13 µm air-gap by surface micromachining technique. To minimize the proximity effect [26], the windings of transformers need to be as short as possible, and the turns need to be spaced apart to separate the signal lines. Therefore, we used the full-wave electromagnetic (EM) commercial software (Ansoft HFSS) to optimize the signal lines (W) and space (S) between each lines, which were 60 and 25 µm, respectively. The inner symmetric transformers contained three differential ground (G), signal (S), ground (G), signal (S) and ground (G) lines, and the widths (S_1_) of each pad were 60 µm. The ground ring structure was used to connect all the ground terminals of this transformer, and the line width (W_1_) was 115 µm. To further approach the characteristic impedance (Z_0_) and to reduce the propagation of the reflected wave at high frequency, we adopted the input/output ports of this power divider with matching MIM capacitors, and the dimensions of the input/output MIM capacitors were 550 (W_c1_) × 550 (l_c1_) µm^2^ and 530 (W_c2_) × 1225 (l_c2_) µm^2^, respectively.

Air, nitride (Si_3_N_4_) [27,28], oxide (SiO2) [29,30] and polymer (SU-8) [31,32] have been used as the material of the insulator of MIM capacitor, but the relative permittivity (~1) of air is too low to achieve the smaller dimensions of capacitors, and nitride and oxide capacitors have higher relative permittivity. In addition, the temperature required for both materials has to be above 200 °C, which leads to difficulty in dislodging the sacrificial layer (AZ 4620) under the insulator layer. So, we chose polymer, which has moderate relative permittivity (~3) and high compatibility, as the material for MIM capacitor.

The inside diameters (ID) and outside diameters (OD) of six-port transformer-based power divider were 345 µm and 760 µm, respectively. The chip dimension including test pads was 2920 × 2780 µm^2^, and Table 1 lists the major design parameters of the proposed six-port transformer-based power divider.

## 3. Fabrication of the Six-Port Transformer-Based Power Divider

**Figure 4:**
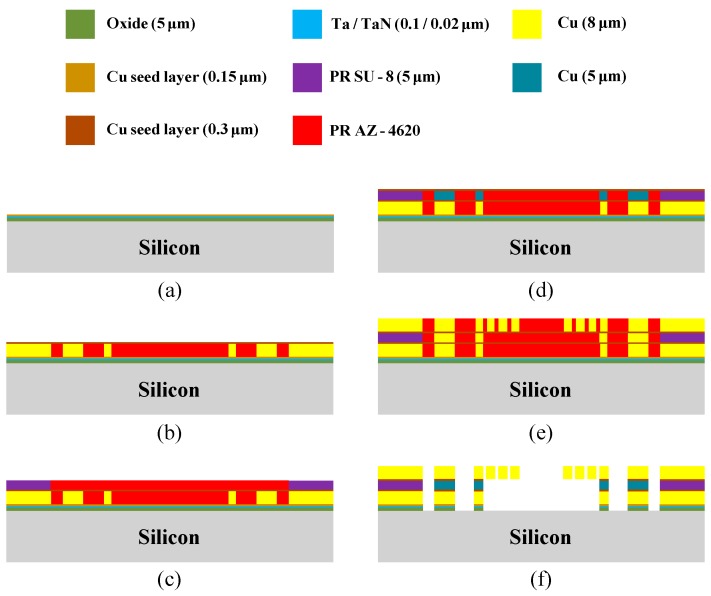
Main processing steps of the proposed power divider: (**a**) 5000/200/1000/1500-Å-thick silicon dioxide/TaN/Ta/Cu multilayers deposition, (**b**) electroplating 8-μm-thick bottom copper conducting layer and 3000-Å-thick copper seed layer deposited, (**c**) 5-μm-thick polymer insulator layer coated and patterning, (**d**) electroplating 5-μm-thick copper supporting layer and 3000-Å-thick copper seed layer deposited, (**e**) electroplating 8-μm-thick top copper symmetric conducting layer, and (**f**) remove the sacrificial and cooper seed layers.

The proposed power divider, whose structure is shown in Figure 4, was fabricated utilizing surface micromachining technology with four photomasks. The 5000-Å-thick silicon dioxide isolation layer was grown on a 525-μm-thick 4-inch silicon wafer using a low-pressure chemical vapor deposition system (LPCVD). A 200-Å-thick TaN, a 1000-Å-thick Ta and a 1500-Å-thick Cu multilayers were continually deposited onto silicon oxide layer by RF-sputter system to enhance the adhesion between silicon oxide and Cu layer (Figure 4a). An 8-μm-thick bottom copper conducting layer structure was patterned and deposited by one photolithography process and one electroplating process, and a 3000-Å-thick copper seed layer was deposited by RF magnetron sputtering (Figure 4b). A 5-μm-thick polymer insulator configuration layer of MIM capacitor on seed layer was coated by spin coater and patterned by wet etching (2.38% C_4_H_13_NO), as shown in Figure 4c. In addition to the insulator layer, a 5-μm-thick copper supporting layer was also deposited and patterned by electroplating system and photolithography process onto the same seed layer and a 3000-Å-thick copper seed layer for electroplating, as illustrated in Figure 4d. Figure 4e depicts the final deposited thin layer, an 8-μm-thick top copper symmetric conducting layer, which was deposited and patterned by electroplating and photolithography process, respectively. The photoresist (AZ 4620) etchant (persulfate) and Cu etchant (persulfate and H_2_SO_4_) were used to remove the sacrificial and cooper seed layers, and Ta/TaN thin layers were etched out in 40 wt % and 90°C KOH etching solution for 30 min to fully release the suspended micro structure. Finally, the whole power divider consisted of six thin-film of various materials, including one 0.5-µm-thick silicon oxide isolation layer, one 0.27-µm-thick TaN/Ta/Cu bottom electrode, 5-µm-thick supporting posts and both of the 8-µm-thick bottom and top conducting layers. The total thickness of the power divider was 21.87 μm.

## 4. Results and Discussion

**Figure 5:**
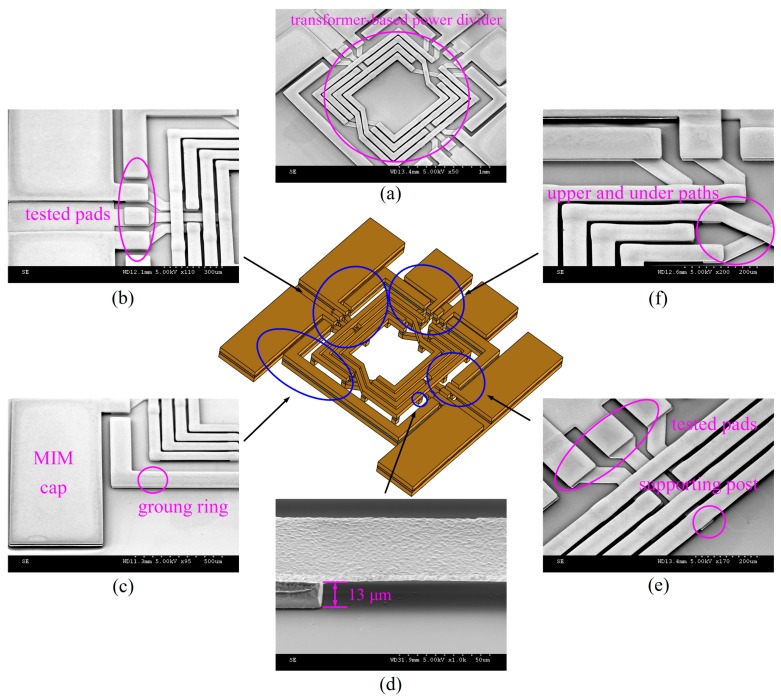
Scanning electron microscope (SEM) optical micrographs: (**a**) free-standing symmetric structure of the six-port power divider, (**b**) GSGSG tested pads, (**c**) MIM capacitor and copper ground ring, (**d**) 13-μm-tall copper supporting post, (**e**) GSGSG tested pads and supporting post, and (**f**) 5-µm air-gap distance from the under path to the upper path.

The major portion and scanning electron microscope (SEM) micrographs (with an enlarged view) of the fabricated six-port transformer-based power divider is shown in Figure 5. Figure 5a presents a free-standing copper symmetric structure well suspended by copper supporting posts on the silicon substrate with a 13-µm air-gap underneath. Figure 5b,e present the output GSGSG tested pads. Figure 5d shows high magnification SEM micrograph of the 13-μm-tall copper supporting post between the Cu suspended structure and the substrate. The MIM capacitor and 21-μm-thick copper ground ring are illustrated in Figure 5c, and 5-µm air-gap distance from the under path to the upper path is shown in Figure 5f.

**Figure 6:**
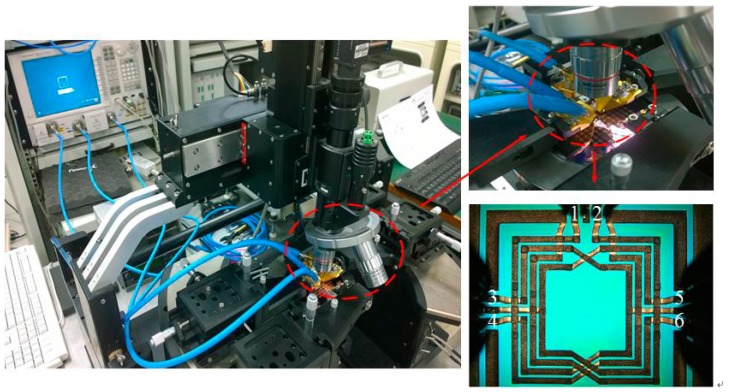
Radio frequency (RF) characteristics measurement environment.

The six-port transformer-based power divider with 2.4 GHz operating frequency was measured on-wafer using an Agilent N5247A to directly obtain the S-parameter data, and the input and output ports were connected with the Cascade Z040-K3N-GSGSG-100 probes. Moreover, the optical image of the micromachined power divider denotes the port numbers for distinguishing the measured S-parameter results, as shown in Figure 6. Since the power divider has six network ports with a differential port considered as two network ports, the power divider is measured between one-side (ports 1, 3 and 4) and the other side (ports 2, 5 and 6) in configurations of three different network ports, whereas the remaining network ports are consistently and simultaneously matched with 50-Ω terminations.

**Figure 7:**
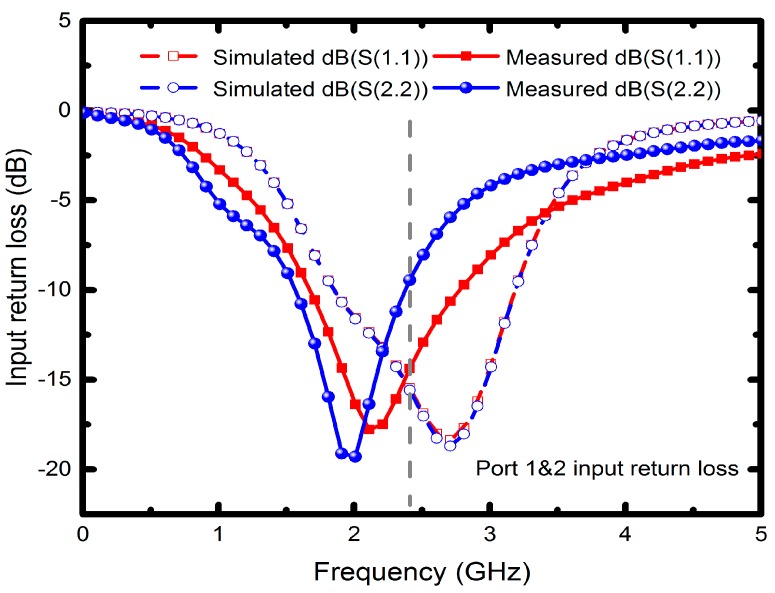
Input return losses of the proposed power divider.

**Figure 8:**
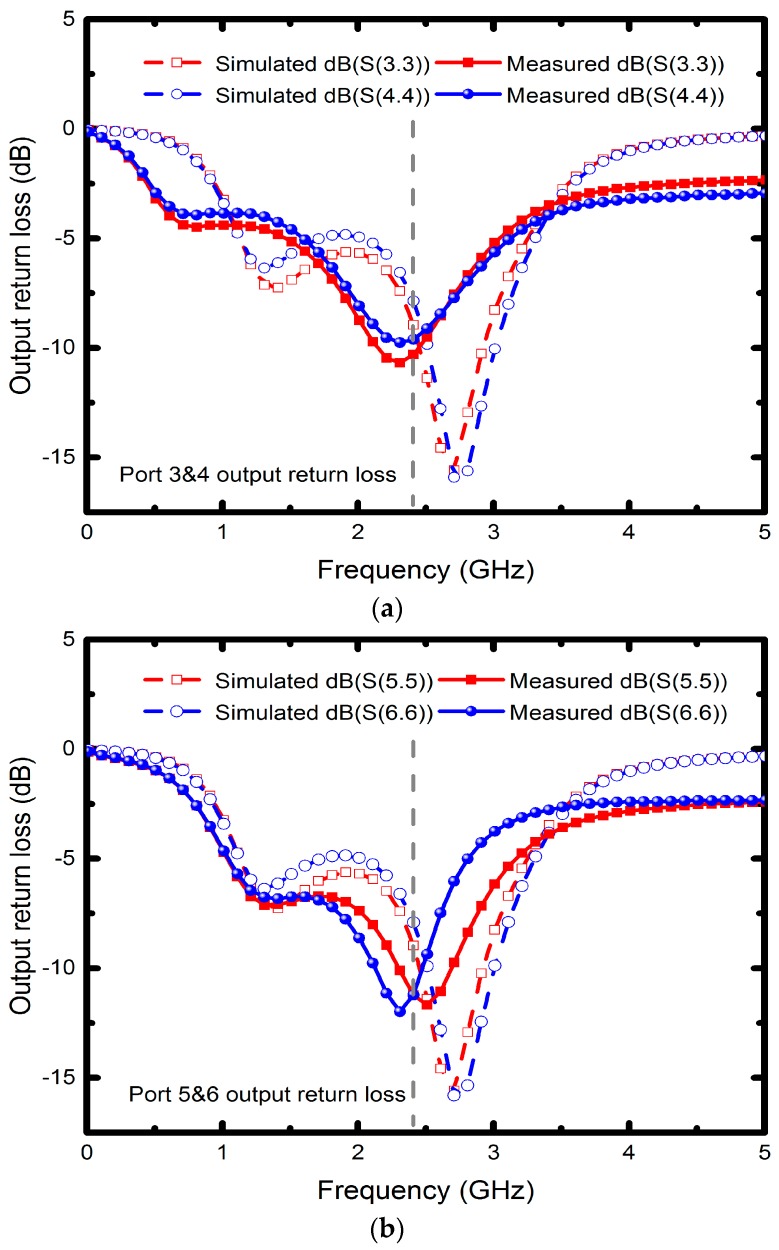
Output return losses of the (**a**) port 3 and 4; (**b**) port 5 and 6.

**Figure 9:**
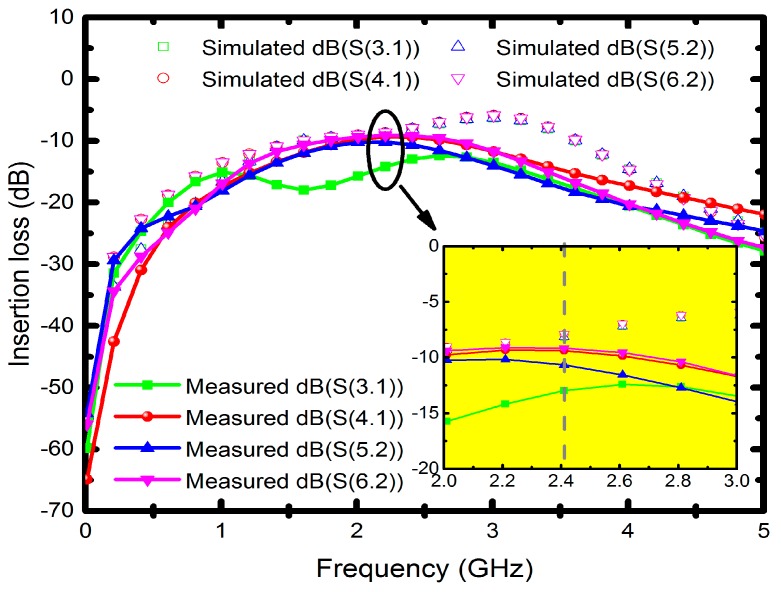
Insertion losses of the six-port power divider.

**Figure 10:**
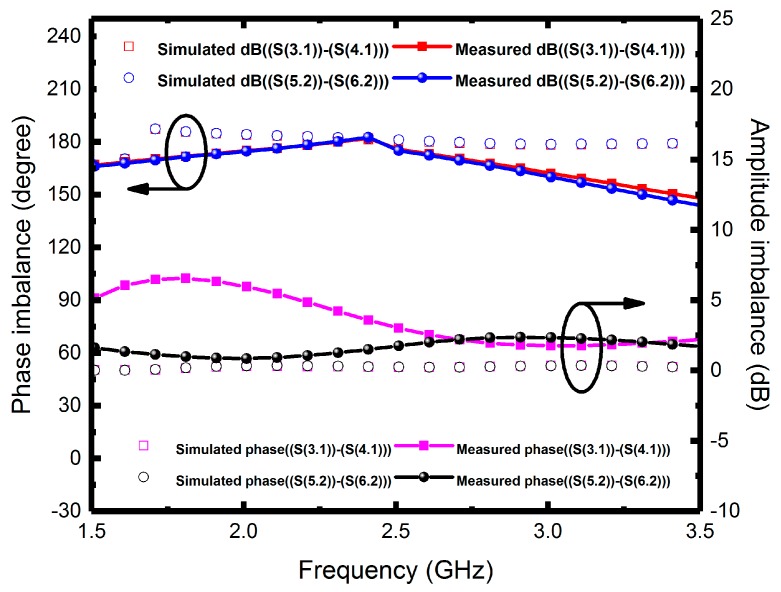
Phase and amplitude imbalances of the six-port power divider.

**Figure 11:**
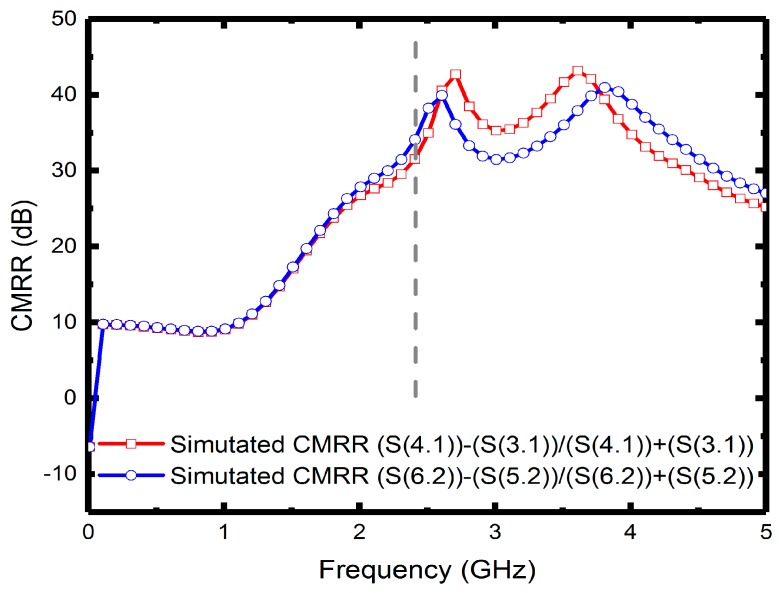
CMRRs of the of the power divider.

Since the measured operating frequency is often lower than simulated frequency, the optimum simulated high-frequency characteristics were set at 2.7 GHz. Figure 7 illustrates the measured input return losses (S11 and S22) with simulation results versus frequency. Although the nadir S11 and S22 are −17.7 dB at 2 GHz and −19.2 dB at 2.1 GHz, the moderate input return losses are around −10 dB at 2.4 GHz. The simulated and measured output return losses (S33, S44, S55 and S66) ranging from 0 to 5 GHz are shown in Figure 8a,b, in which all the simulated performances are below −15 dB at 2.7 GHz. The measured output return losses are beneath −10 dB and are close to the operating frequency. The frequency difference of input and output return losses between simulated and measured frequency is caused by the material, copper, which may undergo quality changes during electroplating. However, in simulation, we set copper as an ideal conductor. Moreover, the operating frequency of the implemented six-port power divider is 2.4 GHz, and it is susceptible to signal interference from other nearby wireless networks and devices when measured in open spaces. Since the proposed divider uses an up-and-down coupler to transmit signals, the first dip of the output return loss was due to the zero point caused by the self-inductance of the divider and the capacitance between the internal metals and the conductor, whereas the lower dip was resulted from the resonance between the internal inductance and external capacitance. The resonant frequency was set as the operating frequency of the power divider. Figure 9 presents the performance of insertion losses (S31, S41, S52 and S62), and the simulated results are about −8 to −6.6 dB from 2.4 to 2.7 GHz. Measured insertion losses of S31, S41, S52 and S62 are −12.9, −9.3, −10.6 and −9.1 dB. The measured average results are lower than −8 dB due to poor input/output matches and some remains such as photoresist or particle that might not be completely removed. Besides, Figure 10 displays the comparison between the measured amplitude and phase imbalances with the simulated ones at frequency range from 1.5 to 3.5 GHz. According to the two output ports (port 3 and 4, port 5 and 6) of the proposed power divider, the minimum amplitude imbalance is 1.5 dB (port 5 and 6), whereas the maximum is below 3.6 dB (port 3 and 4). Phase imbalances are both less than 1°, which denotes moderate amplitude imbalance but excellent phase imbalance output at 2.4 GHz operating frequency. Moreover, there is an important characteristic of common-mode rejection ratio (CMRR) indicating the performance of differential device, and the simulated results reveal that both the CMRRs are larger than 30 dB, as shown in Figure 11.

Based on the nearby operating frequency, Table 2 tabulates a performance comparison between the proposed six-port transformer-based power divider and prior state-of-the-art approaches. The power divider with two single-to-differential paths than the other literatures. Our design also attains a phase imbalance of 1° and chip area of 2.9 × 2.8 mm^2^. Although the prior power splitters in [16,33] exhibit a lower amplitude imbalance, they consume a larger chip area. It is obvious that the proposed power divider has a good phase imbalance.

## 5. Conclusions

We have proposed a suspended six-port transformer-based power divider with two simultaneously sampled single-ended input and differential-ended outputs for multiport transceivers. With low input/output return losses (S11, S22, S33, S44, S55 and S66), which are −10 dB, minimum transmission imbalance (1.5 dB) and excellent phase imbalance output (1°), the realized six-port power divider is well suited for complex wireless networks and applications.

## Figures and Tables

**Table 1:** The parameters of the proposed power divider.

Parameter	w	s	S_1_	W_1_	W_c1_	l_c1_	W_c2_	l_c2_	ID	OD
(μm)	60	25	60	115	550	550	530	1225	345	760

**Table 2:** Performance comparison.

Reference	16	19	33	This Work
Technology	Microstrip Line	Microstrip Line and MEMS	Microstrip Line	MEMS
Single-to-diff. path	1	1	1	2
Operating frequency (GHz)	2	2.5	2.1	2.4
Return loss (dB)	N/A	N/A	−15	−10
Amplitude imbalance (dB)	1.3	2.4	0.6	1.5
Phase imbalance (degree)	5.24	5	N/A	1
Chip area (mm^2^)	117.2 × 37.7	20 × 16	83.8 × 102.3	2.9 × 2.8

## References

[1] Chung Y.H., Liao C.H., Lin C.W., Shih Y.S., Li C.F., Hung M.H., Liu M.C., Wu P.A., Hsu J.L., Hsu M.Y. A dual-band 802.11abgn/ac transceiver with integrated PA and T/R switch in a digital noise controlled SoC. Proceedings of the 2015 IEEE Custom Integrated Circuits Conference (CICC).

[2] Ramella M., Fabiano I., Manstretta D., Castello R. A 2.4 GHz low-power SAW-less receiver for SoC coexistence. Proceedings of the 2015 IEEE Asian Solid-State Circuits Conference (A-SSCC).

[3] Wickert M., Mayer U., Ellinger F. (2012). 802.11a compliant spatial diversity receiver IC in 0.25-μm BiCMOS. IEEE Trans. Microw. Theory Tech..

[4] Gomez-Calero C., Cuellar L., de Haro L., Martinez R. A 2 × 2 novel MIMO testbed for DVB-T2 systems. Proceedings of the 2009 IEEE International Symposium on Broadband Multimedia Systems and Broadcasting.

[5] Chien G., Leong P.B., Son S.W., Tsai M., Tse L. A fully-integrated dual-band MIMO transceiver IC. Proceedings of the IEEE Radio Frequency Integrated Circuits (RFIC) Symposium.

[6] Zhang L., Zhu X., Chen P., Tian L., Zhai J. Broadband four-way power divider for active antenna array application. Proceedings of the 2013 International Symposium on Antennas & Propagation.

[7] Gan H., Wong S.S. Integrated transformer baluns for RF low noise and power amplifiers. Proceedings of the IEEE Radio Frequency Integrated Circuits (RFIC) Symposium.

[8] EL-Gharniti O., Kerherve E., Begueret J.B. A 5 GHz Low Noise Amplifier with On-Chip Transformer-Balun. Proceedings of the 2006 European Conference on Wireless Technology.

[9] Yeh J.F., Yang C.Y., Kuo H.C., Chuang H.R. A 24-GHz transformer-based single-in differential-out CMOS low-noise amplifier. Proceedings of the 2009 IEEE Radio Frequency Integrated Circuits Symposium.

[10] Long J.R. (2000). A low-voltage 5.1-5.8-GHz image-reject downconverter RF IC. IEEE J. Solid-State Circuits.

[11] Vorst D.V., Mirabbasi S. Low-power 1V 5.8 GHz bulk-driven mixer with on-chip balun in 0.18 mmCMOS. Proceedings of the 2008 IEEE Radio Frequency Integrated Circuits Symposium.

[12] Syu J.S., Lu H.L., Meng C. (2012). A 0.6-V 30 GHz CMOS quadrature VCO using microwave 1:1:1 trifilar transformer. IEEE Microw. Wirel. Compon. Lett..

[13] Lee S., Lee S., Park H., Kim W., Kwon H., Jeong J., Kwon Y. (2016). W-band dual-channel receiver with active power divider and temperature-compensated circuit. Electron. Lett..

[14] Weng S.H., Chang H.Y. (2014). A Broadband inductorless active power divider for 10 Gbps high speed transmissions. IEEE Microw. Wirel. Compon. Lett..

[15] Lan X., Lu X., Blumenthal T., Fratello V., Chan W., Truong M., Kiyono K., Zhang Y., Gu G., Tan M. Ultra-wideband microwave components fabricated using low-cost aerosol-jet printing technology. Proceedings of the 2015 IEEE Radio and Wireless Symposium (RWS).

[16] Zhang W., Liu Y., Wu Y., Shen J., Li S., Yu C., Gao J. (2014). Anovel planar structure for implementing power divider or balun with variable power division. Prog. Electromagn. Res. C.

[17] Trantanella C.J. A novel power divider with enhanced physical and electrical port isolation. Proceedings of the 2010 IEEE MTT-S International Microwave Symposium.

[18] Li C., Lok L.B., Khalid A., Papageorgiou V., Grant J., Cumming D.R.S. A coplanar ring power divider with high isolation for V-band and W-band applications. Proceedings of the 2012 42nd European Microwave Conference.

[19] Huang C., Chen D., Leidich S., Gessner T. (2010). Compact meta-material transmission line balun based on meander lines structure and {MEMS} technology. Microelectron. Eng..

[20] Hua D., Liao X. (2011). X-band coplanarWilkinson power divider based on GaAsMMIC technology. Electron. Lett..

[21] Abaei E., Aviles H.M., Shamsafar A., Boccia L., Amendola G. Optimum design of a miniaturized onchip wide band power divider-combiner combined with impedance transformer. Proceedings of the 2015 9th European Conference on Antennas and Propagation (EuCAP).

[22] Lee H.L., Kim D.Z., Tae H.S., Lee M.Q., Yu J.W. A balanced antenna design for six-port receiver architecture. Proceedings of the 40th European Microwave Conference.

[23] Penirschke A., Mahn T., Angelovski A., Jakoby R. Energy beam position monitor design utilizing grounded coplanar waveguide transmission line pickups and a modified six-port discriminator for particle accelerators. Proceedings of the 2015 IEEE International Instrumentation and Measurement Technology Conference (I2MTC).

[24] Ercoli M., Dragomirescu D., Plana R. Small size high isolation Wilkinson power splitter for 60 GHz wireless sensor network applications. Proceedings of the 2011 IEEE 11th Topical Meeting on Silicon Monolithic Integrated Circuits in RF Systems.

[25] Wheeler H.A. (1942). Formulas for the Skin Effect. Proc. IRE.

[26] Cooke C.M. Proximity effect of a conducting plane in electro-optic field probe measurements. Proceedings of the Conference on Electrical Insulation & Dielectric Phenomena—Annual Report 1983.

[27] Babcock J.A., Balster S.G., Pinto A., Dirnecker C., Steinmann P., Jumpertz R., El-Kareh B. (2001). Analog characteristics of metal-insulator-metal capacitors using PECVD nitride dielectrics. IEEE Electron Device Lett..

[28] Ng C.H., Chew K.W., Chu S.F. (2003). Characterization and comparison of PECVD silicon nitride and silicon oxynitride dielectric for MIM capacitors. IEEE Electron Device Lett..

[29] Kim S.J., Cho B.J., Li M.F., Ding S.J., Zhu C., Yu M.B., Narayanan B., Chin A., Kwong D.L. (2004). Improvement of voltage linearity in high-/spl kappa/MIM capacitors using HfO_2_-SiO_2_ stacked dielectric. IEEE Electron Device Lett..

[30] Robertson J. (2004). High dielectric constant oxides. Eur. Phys. J. Appl. Phys..

[31] Ghannam A., Viallon C., Bourrier D., Parra T. Dielectric microwave characterization of the SU-8 thick resin used in an above IC process. Proceedings of the 2009 European Microwave Conference (EuMC).

[32] Pierce R.G., Islam R., Henderson R.M., Blanchard A. (2014). SU-8 2000 millimeter wave material characterization. IEEE Microw. Wirel. Compon. Lett..

[33] Kalyan R., Rawat K., Koul S.K. Design of reconfigurable concurrent dual-band quarter-wave transformer with application of power combiner/divider. Proceedings of the 2015 IEEE MTT-S International Microwave and RF Conference (IMaRC).

